# Occlusal Sensitivity to Articulating Foils in Patients With Temporomandibular Disorders and Healthy Controls

**DOI:** 10.1111/joor.70164

**Published:** 2026-02-12

**Authors:** Iva Z. Alajbeg, Marko Zlendić, Iva Biloš, Ivan Boras, Ema Vrbanović Đuričić, Ivan Alajbeg

**Affiliations:** ^1^ Department of Removable Prosthodontics University of Zagreb School of Dental Medicine Zagreb Croatia; ^2^ Department of Dental Medicine University Hospital Centre Zagreb Zagreb Croatia; ^3^ University of Zagreb School of Dental Medicine Zagreb Croatia; ^4^ Department of Oral Medicine University of Zagreb School of Dental Medicine Zagreb Croatia

**Keywords:** chronic pain, dental occlusion, signal detection theory, temporomandibular disorders, touch perception

## Abstract

**Objectives:**

To compare occlusal tactile acuity (OTA), the ability to detect and discriminate occlusal contacts, between patients with chronic painful temporomandibular disorders (TMDp) and healthy controls and to examine the influence of pain and oral behaviours on foil perception.

**Methods:**

Forty TMDp patients (34 female, 6 male) and 40 healthy controls (20 female, 20 male) completed OTA testing with foils 8–56 μm thickness and a sham stimulus (48 trials). Participants bit in maximal intercuspal position and gave yes/no responses regarding foil perception. Oral behaviours were assessed using the Oral Behaviour Checklist (OBC). Repeated‐measures ANOVA evaluated group and thickness effects, and signal detection theory separated perceptual sensitivity from response bias.

**Results:**

TMDp patients showed higher recognition accuracy across thin foil thicknesses, with the most robust group difference observed at 32 μm after correction for multiple comparisons. They also exhibited higher perceptual sensitivity and a more liberal response criterion. Regression analyses identified TMDp status and female sex as positive predictors of perceptual sensitivity, whereas OBC‐total and OBC‐night were negative predictors. Models with decision bias as the outcome showed that TMDp predicted a more liberal criterion, while all OBC subscales predicted a more conservative criterion.

**Conclusion:**

TMDp participants detected thinner foils more accurately but tended to overreport contact presence. In contrast, frequent oral behaviours were associated with reduced perceptual sensitivity and more conservative response tendencies.

## Graphical abstract

Using articulating foils of varying thickness, occlusal tactile acuity was compared between patients with chronic painful temporomandibular disorders (TMDp) and healthy controls. TMDp patients showed enhanced detection of thinner foils and greater perceptual sensitivity, accompanied by a more liberal decision strategy, whereas higher frequencies of oral behaviours were linked to reduced sensitivity and conservative response patterns.

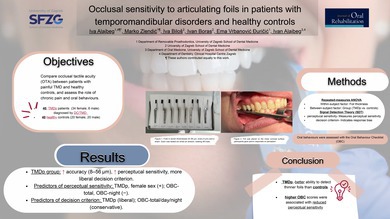

## 1 Introduction

Sensory information from proprioceptors in the periodontal ligament (PDL), masticatory muscles, and temporomandibular joints (TMJ) is transmitted via afferent fibres of the trigeminal nerve to the trigeminal brainstem sensory nuclei and thalamus, before projecting to the cerebral cortex, enabling occlusal tactile acuity (OTA) [1]. This sensory ability to detect occlusal contacts and perceive them as distinct plays a key physiological role in mastication, gustation, and swallowing [2]. The activity of these sensory pathways may be influenced by behavioural, emotional, and biological factors, including nociplastic pain, defined as “pain that arises from altered nociception despite no clear evidence of actual or threatened tissue damage” [3]. This type of pain is considered a characteristic feature of chronic painful temporomandibular disorders (TMDp). Psychological factors, such as anxiety and pain catastrophising, are known to influence OTA. Although increased somatic awareness might be expected to enhance occlusal perception, previous studies have reported contradictory results. In healthy adults, increased somatosensory amplification and hypervigilance appear to diminish the perception of thin foils [4, 5]. A similar effect on the decrease of OTA in healthy adults has been observed after inducing acute pain on the PDL with orthodontic separators [6].

Beyond experimentally induced pain, paradoxical discrepancies emerge when OTA results are compared with quantitative sensory testing (QST) outcomes. The minimal interdental threshold evaluation showed that pain‐free individuals reached 81%–100% correct responses with the 24‐μm foil. In contrast, the TMDp subgroups with myalgia and arthralgia required the 30‐μm foil to achieve the same level of perceptiveness [7]. These findings contradict QST results, which have demonstrated significantly lower pressure and pain thresholds in TMDp myalgia patients compared with healthy controls [8]. However, another study showed that the TMD group had a lower occlusal tactile threshold, indicating increased OTA and a greater ability to perceive thinner foils [9]. Similarly, in another chronic orofacial pain disorder, burning mouth syndrome, patients also exhibited higher OTA than controls [10]. Altered OTA may help explain certain clinical phenomena, such as phantom bite, which is characterized by persistent, non‐verifiable occlusal discrepancies and substantially impacts quality of life [11].

Interestingly, although TMDp patients report higher levels of anxiety and sensory amplification, which in pain‐free individuals are typically associated with reduced OTA, chronic pain appears to facilitate OTA rather than diminish it [12]. Behavioural factors, such as the frequency of oral behaviours, may also influence OTA. Healthy individuals with a higher frequency of oral behaviours are more perceptive of foils than those with a lower frequency [13]. Repeated occlusal testing with aluminium foils has increased OTA, suggesting a potential link with the oral behaviour frequency [14]. Conversely, an inverse relationship is also plausible. Sustained or repetitive stimulation of periodontal proprioceptors may induce sensory adaptation and alter afferent input, reducing receptor responsiveness and diminishing perceptual sensitivity to subtle occlusal stimuli. Although an association between TMDp and more frequent oral behaviours has been reported, its relationship with OTA remains unclear [15].

Therefore, this study sought to address this knowledge gap by comparing OTA between patients with chronic painful TMD and healthy controls and by examining how chronic pain and oral behavioural habits influence the perception of articulating foils of varying thicknesses.

## 2 Materials and Methods

### 2.1 Study Protocol

This case–control study was conducted at the University of Zagreb School of Dental Medicine (Zagreb, Croatia). The study protocol followed the ethical principles of the Declaration of Helsinki and was designed in accordance with the STROBE guidelines [16]. Ethical approval was obtained from the Ethics Committee of the School of Dental Medicine, University of Zagreb (05‐PA‐30‐22‐11/2023), and the trial was registered as NCT07163494 on ClinicalTrials.gov on September 8, 2025.

Data collection occurred between June and September 2025. During this period, 125 patients presenting with orofacial pain or discomfort were examined by calibrated and experienced orofacial pain experts (IZA and MZ) at the Department of Removable Prosthodontics. Of these, 40 patients met the inclusion criteria and agreed to participate in the study. A control group of 40 healthy individuals, mainly employees and students of the School of Dental Medicine, was also recruited. All participants were of Middle or Southern European origin and provided written informed consent after receiving detailed study information.

### 2.2 Study Participants

The case group comprised 40 participants (34 female, 6 male) diagnosed with chronic painful temporomandibular disorders (TMDp). Inclusion criteria were as follows: pain in the temporomandibular joint and/or masticatory muscles persisting or recurring for more than 6 months; spontaneous pain > 30 mm on the Numerical Pain Rating Scale (NPRS) at the first examination; and a diagnosis of arthralgia and/or myalgia according to the Diagnostic Criteria for Temporomandibular Disorders (DC/TMD) [17]. Inter‐rater reliability for DC/TMD diagnoses was established prior to recruitment through joint calibration sessions (κ = 0.86), demonstrating excellent agreement.

Exclusion criteria included the following: loss of posterior teeth affecting occlusal support; removable dentures or fixed prosthodontics; periodontal disease; endodontically treated teeth; active caries; acute pain (< 3 months); history of head or neck trauma; headache unrelated to TMD (per International Classification of Headache Disorders, ICDH II); pain due to fibromyalgia; systemic diseases; diagnosed psychiatric disorders; and history or current misuse of analgesic medication.

The control group (CTR) comprised 40 healthy participants (20 female, 20 male) with complete natural dentition, no history of chronic pain disorders, and no current use of analgesic medication.

### 2.3 Data Collection

At the initial examination, data were collected through a structured anamnesis, including reason for seeking care, pain characteristics (duration, intensity, and type), functional limitations of the lower jaw, current pharmacotherapy, and awareness of oral behavioural habits.

The clinical examination comprised palpation of the masticatory muscles, TMJs, and submandibular and retromandibular regions, as well as measurement of mandibular movements (pain‐free mouth opening, unassisted and assisted mouth opening, lateral excursions, and protrusion) and assessment of TMJ sounds (presence and characteristics) [18].

All participants completed the validated Croatian version of the Oral Behaviour Checklist (OBC), from which total, daytime, and nocturnal scores were derived [19, 20]. In addition, TMDp patients completed the Graded Chronic Pain Scale (GCPS) and the Jaw Functional Limitation Scale (JFLS). The control group completed only the OBC [21].

### 2.4 Assessment of Occlusal Tactile Acuity

OTA was assessed after participants completed self‐reported questionnaires. The procedure was conducted in a quiet, isolated room. Participants wore headphones and an eye mask to minimise external stimuli, and the buccal mucosa was retracted with a dental mirror during trials.

Assessment of OTA was conducted using seven articulating foils with thicknesses ranging from 8 μm to 56 μm in 8‐μm increments, along with one sham test without a foil. Each foil was placed between the right maxillary second premolar and first molar, and participants were instructed to put their back teeth completely together each time a foil was placed. The right maxillary premolar–molar region was selected for foil placement to ensure a standardised and reproducible contact site across participants with intact posterior occlusion. This site provided stable intercuspation and was unaffected by dental restorations in all participants. The testing site was selected independently of the side of reported pain, and participants with asymmetric posterior occlusal support or missing posterior teeth were excluded. This approach was used to minimise potential laterality bias, given that occlusal tactile acuity primarily reflects central sensory processing rather than side‐specific pathology. Each thickness was tested six times in random order, resulting in 48 trials per participant.

Following each trial, participants were asked whether they perceived a foil between their teeth (“yes” or “no”). Responses were recorded in Google Forms and subsequently inserted into an Excel worksheet. All trials were performed by the same investigator (BI), while another researcher selected foils and recorded the answers (IB). Examiner blinding was maintained by assigning separate roles: one examiner randomly selected the foil thickness and recorded participants' responses, while another, blinded to foil thickness, placed the foils intraorally. This two‐operator design ensured independence between stimulus delivery and response recording.

### 2.5 Statistical Analysis

Sample size was estimated a priori from published OTA data. Using TMDp case–control results that detected a 13‐percentage‐point between‐group difference (SD = 10%) with Bonferroni‐adjusted α = 0.005 and 80% power, the required size was 18 participants per group. To allow for potential attrition and unusable data, we planned to recruit 40 participants per group, providing a conservative margin beyond the calculated minimum. As a sensitivity check, planning for a medium–large standardised effect (d≈0.67) would have required ~35 per group at α = 0.05 (two‐sided), which is consistent with our final sample.

Data were analysed using commercial software IBM SPSS 22 (IBM, Armonk, New York, USA). Normality of the measured variables was assessed with the Kolmogorov–Smirnov and Shapiro–Wilk tests. Descriptive statistics were calculated for sociodemographic and clinical variables, with group comparisons performed using independent‐samples *t*‐tests for continuous data and χ^2^ tests for categorical variables.

OTA was evaluated with repeated‐measures ANOVA, with foil thickness (0–56 μm, including sham) as the within‐subject factor and group (TMDp vs. controls) as the between‐subject factor. Significant interactions were followed up by independent‐samples *t*‐tests at individual foil thicknesses. To control for inflated Type I error due to multiple comparisons, Bonferroni correction was applied to the alpha level (0.05 divided by the number of *post hoc* tests).

The mean percentage of correct responses was computed for each thickness within each group, following previous approaches to OTA analysis [6, 9]. As clinically interpretable threshold endpoints, MIT50 was defined as the smallest foil thickness (μm) associated with ≥ 50% accuracy, and MIT70 as the smallest thickness with ≥ 70% accuracy. Group differences in MIT50 and MIT70 were compared using χ^2^ tests.

Perceptual sensitivity (*d'*) and decision criterion (*c*) were derived according to signal detection theory. For each participant, the hit rate (HR) was calculated from real–foil trials and the false‐alarm rate (FAR) from sham trials. A log‐linear correction was applied to avoid boundary estimates:

$$\begin{array}{c} \mathrm{HR}=\left(\text{Hits}+0.5\right)/\left(\text{SignalTrials}+1\right),\\ \;\mathrm{FAR}=\left(\text{FalseAlarms}+0.5\right)/\left(\text{NoiseTrials}+1\right). \end{array}$$

$$\begin{array}{c} \text{Z‐scores were obtained by transforming hit and false}\;\\ \text{‐}\;\text{alarm rates into standard normal deviates using the inverse}\\ \text{cumulative distribution function},\text{and the indices were calculated}\;\mathrm{as}:\\ \;d^{'}=Z\left(\mathrm{HR}\right)-Z\left(\mathrm{FAR}\right),\;c=-0.5\left[Z\left(\mathrm{HR}\right)+Z\left(\mathrm{FAR}\right)\right] \end{array}$$

Between‐group comparisons of *d'* and *c* were initially performed using independent‐samples *t*‐tests.

To obtain adjusted estimates, multivariable linear regression models were fitted separately for *d'* and *c*. Group, OBC scores, sex, and age were included as predictors, with age and sex entered as covariates. Due to collinearity, OBC‐total, waking‐state OB, and sleep‐related OB were analysed in separate models. Continuous predictors were mean‐centred and standardised before entry. Prespecified group × OBC interactions were examined but not retained when non‐significant. Results are reported as standardised β coefficients with 95% confidence intervals. Model assumptions were verified through residual diagnostics and multicollinearity checks (VIF < 5). Sex and age were included as covariates in all multivariable regression analyses to account for potential confounding effects.

## 3 Results

### 3.1 Participants' Characteristics

The sample consisted of 54 women (67.5%) and 26 men (32.5%), aged 37.9 ± 15.4 years.

The control group comprised 40 individuals (20 women and 20 men), with an average age of 37.3 ± 16.4 years.

The TMDp group included 34 women (85%) and 6 men (15%) with an average age of 38.5 ± 14.5 years.

All patients reported persistent TMD‐related pain for at least 6 months, meeting the DC/TMD criterion for chronic pain. The average pain intensity at the consultation was 4.6 on the GCPS (minimum: 3; maximum: 9). The average number of days of pain in the last 6 months was 90.9 ± 71.3 days, with over 60% reporting TMD‐related pain in multiple areas. None of the patients were undergoing active occlusal or kinesiotherapy treatment during testing. Occasional use of non‐prescription analgesics was permitted, whereas individuals using antidepressants, anticonvulsants, or muscle relaxants were excluded.

According to the GCPS score, we obtained the following results:

• 3 TMDp patients (3.8%) reported severely limiting.

• 5 TMDp patients (6.3%) had moderately limiting.

• 11 TMDp patients (13.8%) had high intensity pain, without disability.

• 21 TMDp patients (26.3%) were labelled as having low intensity pain, without disability.

Among all TMDp subjects, 23 individuals (57.5%) had more than one diagnosis. Arthralgia was the sole pain diagnosis in 8 participants (20%), while 9 individuals (22.5%) had myalgia as the sole pain diagnosis. In total, myalgia—whether localised, myofascial, or with referral—was found in 80% of the group (32 individuals), and arthralgia was present in 77.5% (31 individuals). Mastication limitation (as measured by JFLS) was present in 75%, mobility limitation in 80%, verbal and emotional expression limitation in 52.5%, and global limitation in 77.5% of TMDp participants.

The OBC showed that individuals with a high frequency of oral parafunctions (OBC total score > 25) were more prevalent in the TMDp group; 85% vs. 45% in the control group.

The frequency of oral behaviours (OBC total score, waking‐state oral behaviours and sleep‐related oral behaviours) was significantly higher in the TMDp group when compared to CTR subjects (32.30 vs. 25.60; 26.97 vs. 22.10; 5.32 vs. 3.50, respectively). When analysing participants depending on sex, no differences in oral behaviour scores (OBC total score, waking‐state oral behaviours, and sleep‐related oral behaviours) were found, both in TMDp and CTR groups (Table 1).

**TABLE 1:** Sociodemographic and clinical characteristics of patients with pain‐related temporomandibular disorders and healthy controls.

Variable			Total	
			CTR (*n* = 40)	TMDp (*n* = 40)
Sex	Female, *n* (%)		20 (50%)	34 (85%)
	Male, *n* (%)		20 (50%)	6 (15%)
	*p* ^a^		**0.001**	
Age	Female	Mean (SD)	34.55 (14.73)	41.02 (14.11)
		*p* ^b^	0.115	
	Male	Mean (SD)	40.52 (17.97)	24.33 (7.12)
		*p* ^b^	0.060	
OBC‐tot	Female	Mean (SD)	27.85 (8.84)^c^	32.21 (7.85)^f^
		*p* ^b^	0.065	
	Male	Mean (SD)	23.35 (9.86)^c^	32.83 (7.14) ^f^
		*p* ^b^	**0.039**	
	Female	Mean (SD)	24.00 (7.04)^d^	27.00 (7.08)^g^
		*p* ^b^	0.013	
Waking‐state oral behaviours	Male	Mean (SD)	20.20 (8.51)^d^	26.83 (6.52) ^g^
		*p* ^b^	0.092	
Sleep‐related oral behaviours	Female	Mean (SD)	3.85 (2.70)^e^	6.00 (1.78)^h^
		*p* ^b^	0.054	
	Male	Mean (SD)	3.15 (2.85)^e^	5.21 (2.29) ^h^
		*p* ^b^	**0.030**	

Abbreviations: TMD‐pain patients – presence of pain disorders including myalgia, arthralgia or both, TMDp; Control group—absence of TMD diagnosis, CTR; Oral Behaviours Checklist, OBC‐tot.

^a^ Chi‐square *t*‐test—between group comparison.

^b^ Independent *t*‐test—between group comparison.

^c^ Independent *t*‐test results—between sex comparison (*p* = 0.137).

^d^ Independent *t*‐test results—between sex comparison (*p* = 0.132).

^e^ Independent *t*‐test results—between sex comparison (*p* = 0.431).

^f^ Independent *t*‐test results—between sex comparison (*p* = 0.856).

^g^ Independent *t*‐test results—between sex comparison (*p* = 0.957).

^h^ Independent *t*‐test results—between sex comparison (*p* = 0.426).

### 3.2 Between‐Group Differences in Occlusal Tactile Acuity

Repeated‐measures ANOVA with foil thickness as the within‐subject factor and group (TMDp vs. controls) as the between‐subject factor revealed a significant main effect of group, F (1,78) = 9.34, *p* = 0.003, indicating that TMDp patients recognised the foils more accurately than controls across conditions. There was also a significant main effect of foil thickness, F (7,546) = 1097.69, *p* < 0.001, showing that recognition accuracy increased with increasing foil thickness. Importantly, a significant group × foil thickness interaction was observed, F (7,546) = 2.51, *p* = 0.015, suggesting that the pattern of recognition across foil thickness levels differed between TMDp patients and controls.

To explore this interaction, *post hoc t*‐tests were conducted for each foil thickness. To control for Type I error, Bonferroni correction was applied to the alpha level (0.05/7 = 0.007). After correction, the between‐group difference remained statistically significant only at 32 μm (*p* = 0.001), while differences at 8 μm (*p* = 0.015), 40 μm (*p* = 0.028), and 56 μm (*p* = 0.049) did not survive the corrected threshold, although they showed the same trend. For the sham test and thicker foils (≥ 48 μm), both groups performed near ceiling level (*p* ≥ 0.50).

**FIGURE 1:**
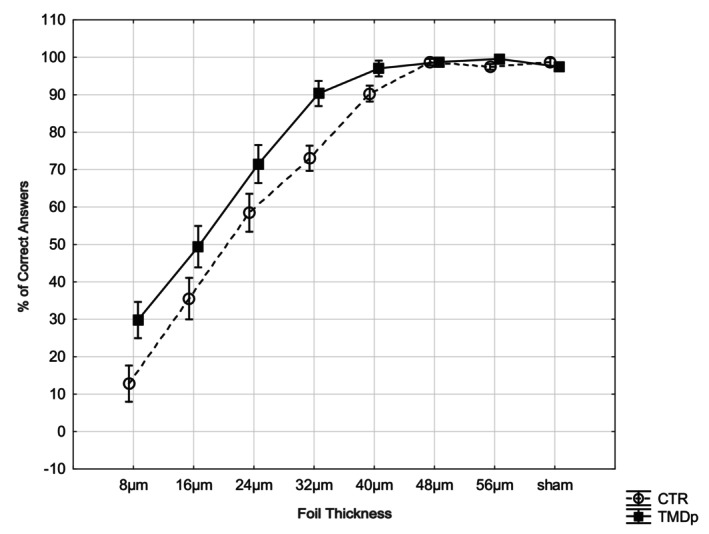
Mean percentage of correct answers for each testing thickness in the TMDp group (continuous line) and in CTR group (dotted line). Participants' answers considered correct whenever “positive” (yes) for foil thicknesses (from 0.008 to 0.0056) and “negative” (no) for sham test (0.000). Error bars represent ± standard errors.

Overall, these results indicate that the group effect was primarily driven by enhanced recognition accuracy of thinner foils in TMDp patients, confirming a distinct perceptual sensitivity profile relative to controls (Figure 1).

### 3.3 MIT Thresholds

**FIGURE 2:**
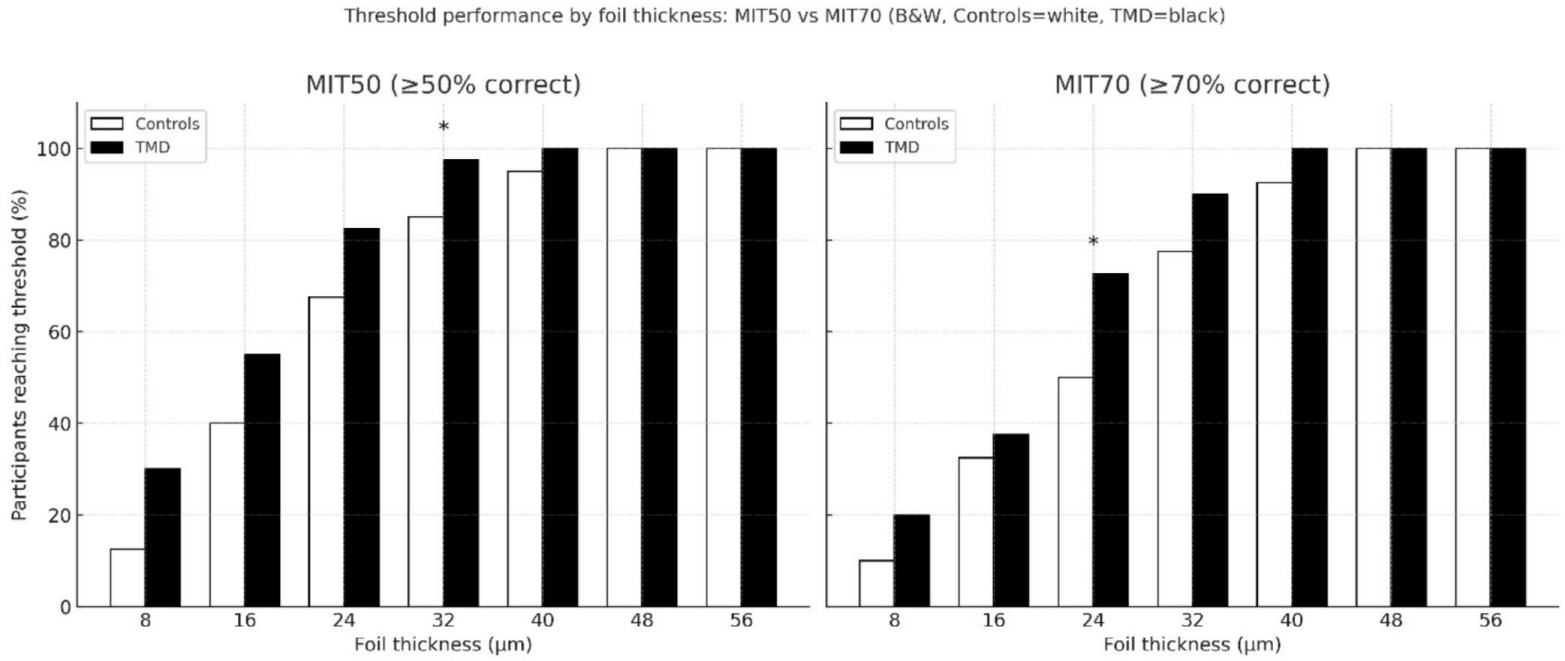
Proportion of participants reaching MIT50 and MIT70 thresholds across foil thicknesses in TMDp and control group. MIT50 and MIT70 denote the minimal identifiable thicknesses at which participants correctly detected foil presence in at least 50% and 70% of trials, respectively.

The proportion of participants reaching the occlusal tactile acuity thresholds increased progressively with foil thickness in both groups (Figure 2). At low thicknesses (8–16 μm), the performance was poor overall, with no significant differences between groups. At intermediate thicknesses, group separation became more apparent. At 24 μm, a significantly greater proportion of TMD participants reached the MIT70 threshold compared with controls (72.5% vs. 50%, χ^2^ *p* = 0.039). At 32 μm, TMD participants were also more likely to reach the MIT50 threshold than controls (97.5% vs. 85%, χ^2^ *p* = 0.048). At higher thicknesses (≥ 40 μm), nearly all participants in both groups reached the thresholds, indicating ceiling effects and no meaningful differences. Sham trials showed similarly low false‐alarm rates in both groups, with no significant differences (*p* = 0.331).

### 3.4 Group Differences in Perceptual Sensitivity and Response Bias

Independent‐samples *t*‐tests revealed significant between‐group differences in both perceptual sensitivity (*d'*) and decision criterion (*c*). TMDp patients showed significantly higher perceptual sensitivity (mean = 2.24) than controls (mean = 1.89), t(78) = −2.83, *p* = 0.006 (Cohen's d = 0.64, indicating a medium‐to‐large effect size). Furthermore, TMDp patients applied a more liberal decision criterion (mean = 0.26) compared to controls (mean = 0.47), t(78) = 2.96, *p* = 0.004 (Cohen's d = 0.66, a medium‐to‐large effect size).

### 3.5 Multivariable Analysis of Signal Detection Measures

Linear regression analyses were conducted with perceptual sensitivity (*d'*) as the dependent variable, controlling for age and sex. In the first step (covariates only), the model explained 9.5% of the variance in *d'*, F (2,77) = 4.05, *p* = 0.021. After adding group (TMDp vs. control) and OBC‐total, the model was significant, F (4,75) = 4.92, *p* = 0.002, explaining 20.0% of the variance (Adj. R^2^ = 0.16). In the adjusted model, both group (β = 0.31, *p* = 0.010) and sex (β = 0.26, *p* = 0.023) were significant predictors, indicating that TMDp patients and females had higher perceptual sensitivity than controls and males, respectively. OBC‐total was a significant negative predictor (β = −0.27, *p* = 0.018), suggesting that higher OBC scores were associated with reduced *d'*. Age was not significantly related to *d'* (β = −0.06, *p* = 0.58).

When waking‐state OBC was entered instead of OBC‐total, the regression model explained 17.2% of the variance in *d'*, F (4,75) = 3.48, *p* = 0.012. Group (β = 0.27, *p* = 0.022) and sex (β = 0.26, *p* = 0.029) remained significant predictors, indicating higher perceptual sensitivity in TMDp patients and females. Waking‐state OBC showed a negative, trend‐level association with *d'* (β = −0.20, *p* = 0.084), suggesting that higher scores might be linked to reduced sensitivity, although this effect did not reach statistical significance. Age was not related to *d'* (β = −0.07, *p* = 0.49).

When sleep‐related oral behaviours (OBC‐night) were entered into the model, explained variance in *d'* increased to 24.2% (Adj. R^2^ = 0.20), F (4,75) = 5.13, *p* = 0.001. In this adjusted model, group (β = 0.34, *p* = 0.004) and sex (β = 0.23, *p* = 0.037) remained significant predictors, with TMDp patients and females showing higher perceptual sensitivity. OBC‐night was a strong negative predictor (β = −0.35, *p* = 0.002), indicating that higher levels of sleep‐related oral behaviours were associated with reduced perceptual sensitivity. Age was unrelated to *d'* (β = 0.03, *p* = 0.81).

Linear regression analyses were conducted with the decision criterion (*c*) as the dependent variable, controlling for age and sex. In the adjusted model, group was a significant predictor (β = −0.39, *p* = 0.001), showing that TMDp patients applied a more liberal decision criterion than controls. OBC‐total was also significant (β = 0.31, *p* = 0.007), indicating that higher OBC scores were associated with a more conservative criterion. Age showed a trend‐level association (β = 0.19, *p* = 0.066), while sex was not related to *c* (β = −0.12, *p* = 0.28).

When waking‐state OBC was entered instead of OBC‐total, the model explained 18.7% of the variance in *c*, F (4,75) = 4.31, *p* = 0.004. Group remained a significant predictor (β = −0.36, *p* = 0.003), confirming that TMDp patients applied a more liberal criterion than controls. Waking‐state OBC also emerged as a significant positive predictor (β = 0.23, *p* = 0.044), indicating that higher scores were associated with a more conservative response criterion. Age showed a borderline positive association (β = 0.21, *p* = 0.050), while sex was not significant (β = −0.11, *p* = 0.32).

When OBC‐night was entered into the model, explained variance in *c* increased to 26.3% (Adj. R^2^ = 0.22), F (4,75) = 6.69, *p* < 0.001. Group was a strong predictor (β = −0.42, *p* < 0.001), indicating that TMDp patients adopted a more liberal criterion compared to controls. OBC‐night was also a strong positive predictor (β = 0.38, *p* < 0.001), showing that higher sleep‐related oral behaviours were associated with a more conservative decision criterion. Age (β = 0.10, *p* = 0.33) and sex (β = −0.08, *p* = 0.43) were not significant (Table 2).

**TABLE 2:** Linear regression analyses predicting perceptual sensitivity (*d'*) and decision criterion (*c*).

Predictor	*d'* (OBC‐tot)	*d'* (waking‐state OB)	*d'* (sleep‐related OB)	*c* (OBC‐tot)	*c* (waking‐state OB)	*c* (sleep‐related OB)
Group (TMDp vs. CTR)	0.31 [0.086, 0.616], *p* = 0.010	0.27 [0.047, 0.578], *p* = 0.022	0.34 [0.128, 0.648], *p* = 0.004	−0.39 [−0.415, −0.109], *p* = 0.001	−0.36 [−0.391, −0.083], *p* = 0.003	−0.42 [−0.432, −0.133], *p* < 0.001
Sex (Female vs. Male)	0.26 [0.046, 0.592], *p* = 0.023	0.26 [0.033, 0.590], *p* = 0.029	0.23 [0.018, 0.545], *p* = 0.037	−0.12 [−0.243, 0.072], *p* = 0.28	−0.11 [−0.242, 0.081], *p* = 0.32	−0.08 [−0.212, 0.092], *p* = 0.43
Age	−0.06 [−0.010, 0.006], *p* = 0.58	−0.07 [−0.011, 0.005], *p* = 0.49	0.03 [−0.007, 0.009], *p* = 0.81	0.19 [0.000, 0.009], *p* = 0.066	0.21 [0.000, 0.009], *p* = 0.050	0.10 [−0.002, 0.007], *p* = 0.33
OBC score	−0.27 [−0.031, −0.003], *p* = 0.018	−0.20 [−0.031, 0.002], *p* = 0.084	−0.35 [−0.124, −0.029], *p* = 0.002	0.31 [0.003, 0.019], *p* = 0.007	0.23 [0.000, 0.019], *p* = 0.044	0.38 [0.021, 0.076], *p* < 0.001
Adj. R^2^	0.16	0.13	0.20	0.18	0.15	0.22
Model F (df)	4.69 (4,75), *p* = 0.002	3.89 (4,75), *p* = 0.006	5.97 (4,75), *p* < 0.001	5.31 (4,75), *p* < 0.001	4.31 (4,75), *p* = 0.004	6.69 (4,75), *p* < 0.001

*Note:* The rows are general predictors, and the columns specify which type of OBC score was used in the model.

Values are standardised regression coefficients (β); 95% confidence intervals for the regression coefficients are shown in brackets, and *p*‐values are reported.

Abbreviations: TMD‐pain patients—presence of pain disorders including myalgia, arthralgia or both, TMDp; Control group—absence of TMD diagnosis, CTR; Oral Behaviours Checklist, OBC‐tot; Oral Behaviours, OB.

## 4 Discussion

The present study compared OTA between patients with chronic painful TMD and healthy controls. It examined how the presence of pain and oral behavioural habits may influence the perception of foils of varying thicknesses. Our results showed that TMDp patients demonstrated enhanced detection of thinner foils and a greater tendency to report the presence of occlusal contact compared with controls.

Our main results revealed significant between‐group differences, with TMDp patients recognising thinner foils more accurately than controls, particularly at 32 μm thicknesses. In addition, TMDp patients showed significantly higher perceptual sensitivity (*d'*) and adopted a more liberal decision criterion (*c*) compared with controls. Regression analyses further indicated that TMDp status and being female were independently associated with greater perceptual sensitivity, whereas both total and sleep‐related OBC scores were negatively associated with *d'*. With the decision criterion as the dependent variable, TMDp status predicted a more liberal *c*, while OBC scores (total, waking‐ and sleep‐related) predicted a more conservative criterion.

The results of this study are consistent with the findings of Bucci et al., who reported increased OTA in TMDp patients compared with healthy controls [9]. Our findings are also in line with several case–control studies employing QST, which demonstrated that patients with TMDp are more sensitive to tactile stimulation of the skin than TMD‐free controls [22, 23]. However, the findings of Kogawa et al. contrast with ours. Although their case group comprised females with chronic TMDp, they reported higher occlusal detection thresholds compared with healthy controls and suggested that impaired OTA may be a consequence of TMDp symptoms [7]. In contrast, our study found that both TMDp status and female sex were associated with increased OTA. This enhancement likely reflects neuroplastic changes in central pain networks. Chronic TMD pain involves altered thalamic and insular processing, resulting in increased gain of sensory input and reduced inhibitory control [3, 24]. These central mechanisms may explain why persistent pain is accompanied by increased intraoral perceptual acuity. However, the observed perceptual enhancement in TMDp likely reflects an interaction between central and peripheral sensitisation mechanisms. Peripheral sensitisation of periodontal and muscular afferents may increase responsiveness to occlusal stimuli and amplify sensory input reaching central processing centres. Interindividual variability in these processes may also be partly genetically driven as recent evidence indicates that genetic factors can influence pain modulation and treatment outcomes in pain‐related TMD patients [25].

By applying Signal Detection Theory (SDT), our study was able to distinguish perceptual sensitivity (*d'*) from response bias (*c*). SDT provides a framework for distinguishing an individual's true ability to discriminate between signal and noise from the strategy they use when deciding whether a signal is present [26]. Using this approach, we found that TMDp patients not only demonstrated higher sensitivity to occlusal foils but also adopted a more liberal decision criterion. This distinction is clinically relevant as an apparent increase in accuracy may in fact reflect a change in response strategy rather than genuine perceptual enhancement. The application of SDT, therefore, offers a more nuanced understanding of tactile perception in chronic pain populations than accuracy measures alone can provide.

Another factor examined was the relationship between OTA and the frequency of oral behaviours. Bucci et al. reported that healthy individuals with a higher frequency of oral behaviours were more perceptive of articulating foils [13]. In our study, however, higher OBC scores were negatively correlated with perceptual sensitivity and positively associated with a conservative decision criterion. This discrepancy may partly be explained by differences in study populations as Bucci's sample consisted exclusively of healthy participants, whereas our study included both TMDp patients and controls. It is also possible that frequent oral behaviours result in chronic stimulation of periodontal and muscular proprioceptors, potentially leading to reduced neural excitability over time. Interaction analyses further showed that TMD status did not significantly moderate the association between oral behaviour frequency and perceptual sensitivity. The negative OBC–OTA relationship was consistent across both groups, suggesting that frequent oral behaviours may be linked to reduced discrimination accuracy irrespective of chronic pain status. Repetitive stimulation of oral proprioceptors may contribute to attenuation of sensory signals. Similar phenomena have been reported in studies using QST, which demonstrated reduced responsiveness of small nerve endings after repeated stimulation [27]. Consequently, repetitive tooth contact could lead to attenuated afferent signalling from periodontal mechanoreceptors or altered central processing of occlusal sensory input. Clarifying this relationship is essential, given that oral behaviours are a common focus in TMDp management.

Investigating occlusal sensitivity in patients with chronic orofacial pain has important clinical implications. Such patients frequently report perceived occlusal disturbances, sometimes attributing them to previous dental treatments. Consequently, assessing OTA before initiating further dental interventions is essential. In clinical practice, awareness of these altered sensory and decision processes can help prevent unnecessary occlusal adjustments in TMDp patients who report subjective bite changes. Instead, clinicians should prioritize pain management, behavioural interventions targeting oral parafunctions, and patient education about the sensory nature of occlusal perception before considering irreversible dental procedures.

This study has several limitations that may affect the generalisability of its findings. First, it was conducted in a single research centre and within one population, which may limit external validity. There was also a discrepancy in sex distribution between the two study groups, with a higher proportion of females in the TMDp group compared to controls (34 vs. 20, respectively). This reflects the well‐documented epidemiological pattern of pain‐related temporomandibular disorders, which predominantly affect women [28]. Although this imbalance could potentially confound group differences, sex was included as a covariate in all regression models, and additional exploratory analyses stratified by sex confirmed that the direction of the effects remained consistent in both sexes. Therefore, the observed enhancement of occlusal tactile acuity in TMDp patients is unlikely to be solely attributable to sex composition. However, the study design precludes conclusions about causality. Although the occlusal tactile acuity task involved repeated trials, potential fatigue or learning effects were minimised through randomised stimulus presentation and interspersed sham trials; moreover, any residual learning effects would be expected to affect both groups similarly and are therefore unlikely to account for the observed between‐group differences. Another limitation is that the frequency of oral behaviours was assessed using a self‐reported questionnaire, which is subject to recall or reporting bias. Future multicentre studies with larger and more diverse samples, longitudinal follow‐up, objective polysomnographic assessment of oral behaviours, and evaluation of treatment effects on OTA are warranted to validate and extend these findings.

## 5 Conclusion

This study demonstrated that patients with chronic painful TMD exhibited enhanced occlusal tactile acuity compared with healthy controls, particularly at intermediate foil thicknesses. Female sex and TMDp status were independently associated with higher perceptual sensitivity, while oral behaviours, especially sleep‐related behaviours, were negatively associated with sensitivity and positively related to conservative response tendencies. These findings suggest that chronic painful TMD is characterised by altered intraoral somatosensory processing and decision strategies, which may contribute to patients' frequent reports of occlusal disturbances. A clearer understanding of these mechanisms may improve clinical assessment and support more tailored management of orofacial pain conditions.

## Author Contributions

I.Z.A. conceptualised and designed the study, analysed and interpreted the data, and drafted and revised the manuscript. M.Z. carried out the research and wrote the manuscript. I.B. carried out the research and made a graphic abstract. I.B. carried out the research and made a graphic abstract. E.V.Đ. critically revised the manuscript. I.A. guided the conceptualisation and design of the research. All authors have read and agreed to the published version of the manuscript.

## Funding

This research was funded by the European Union – *NextGenerationEU,* through the *National Recovery and Resilience Plan (NPOO*) within the project “Laboratory Investigation of Homeostatic and Pathophysiological Processes in the Orofacial Region_LIPOR” (grant number SFZG‐07‐2025).

## Conflicts of Interest

The authors declare no conflicts of interest.

## Data Availability

The data that support the findings of this study are available from the corresponding author upon reasonable request.

## References

[1] B. J. Sessle , “The Neurobiology of Facial and Dental Pain: Present Knowledge, Future Directions,” Journal of Dental Research 66, no. 5 (1987): 962–981. 3301935 10.1177/00220345870660052201

[2] K. D. Foster , J. M. Grigor , J. N. Cheong , M. J. Yoo , J. E. Bronlund , and M. P. Morgenstern , “The Role of Oral Processing in Dynamic Sensory Perception,” Journal of Food Science 76, no. 2 (2011): 49–61. 10.1111/j.1750-3841.2010.02029.x 21535784

[3] C. M. Kaplan , E. Kelleher , A. Irani , A. Schrepf , D. J. Clauw , and S. E. Harte , “Deciphering Nociplastic Pain: Clinical Features, Risk Factors and Potential Mechanisms,” Nature Reviews. Neurology 20, no. 6 (2024): 347–363. 38755449 10.1038/s41582-024-00966-8

[4] G. Coppola , A. Michelotti , V. Simeon , M. Koutris , F. Lobbezoo , and R. Bucci , “Association Between Psychological Traits and Occlusal Tactile Acuity of Healthy Individuals,” Journal of Oral Rehabilitation 51, no. 11 (2024): 2452–2459. 39209765 10.1111/joor.13828

[5] N. Sadeghlo , J. Selvanathan , D. Koshkebaghi , and I. Cioffi , “Aberrant Occlusal Sensitivity in Adults With Increased Somatosensory Amplification: A Case‐Control Study,” Clinical Oral Investigations 28, no. 5 (2024): 250. 38613726 10.1007/s00784-024-05628-z

[6] R. Bucci , M. Koutris , V. Simeon , F. Lobbezoo , and A. Michelotti , “Effects of Acute Pain and Strain of the Periodontium due to Orthodontic Separation on the Occlusal Tactile Acuity of Healthy Individuals,” Clinical Oral Investigations 25, no. 12 (2021): 6833–6840. 33954851 10.1007/s00784-021-03971-z PMC8602128

[7] E. M. Kogawa , P. D. Calderon , J. R. Lauris , L. F. Pegoraro , and P. C. Conti , “Evaluation of Minimum Interdental Threshold Ability in Dentate Female Temporomandibular Disorder Patients,” Journal of Oral Rehabilitation 37, no. 5 (2010): 322–328. 20180897 10.1111/j.1365-2842.2010.02062.x

[8] H. Meng , J. Dai , and Y. Li , “Quantitative Sensory Testing in Patients With the Muscle Pain Subtype of Temporomandibular Disorder: A Systemic Review and Meta‐Analysis,” Clinical Oral Investigations 25, no. 12 (2021): 6547–6559. 34487241 10.1007/s00784-021-04171-5

[9] R. Bucci , M. Koutris , S. Palla , G. F. Sepúlveda Rebaudo , F. Lobbezoo , and A. Michelotti , “Occlusal Tactile Acuity in Temporomandibular Disorder Pain Patients: A Case‐Control Study,” Journal of Oral Rehabilitation 47, no. 8 (2020): 923–929. 32433776 10.1111/joor.12996

[10] F. Canfora , D. Adamo , R. Rongo , et al., “Occlusal Tactile Acuity in Patients With Burning Mouth Syndrome: A Case‐Control Study,” Seminars in Orthodontics 30, no. 3 (2024): 329–334.

[11] T. T. H. Tu , M. Watanabe , G. K. Nayanar , et al., “Phantom Bite Syndrome: Revelation From Clinically Focused Review,” World Journal Psychiatry 11, no. 11 (2021): 1053–1064. 10.5498/wjp.v11.i11.1053 PMC8613755 34888173

[12] E. Vrbanović , M. Zlendić , and I. Z. Alajbeg , “Association of Oral Behaviours' Frequency With Psychological Profile, Somatosensory Amplification, Presence of Pain and Self‐Reported Pain Intensity,” Acta Odontologica Scandinavica 80, no. 7 (2022): 522–528. 35254961 10.1080/00016357.2022.2042380

[13] R. Bucci , M. Koutris , F. Lobbezoo , and A. Michelotti , “Occlusal Sensitivity in Individuals With Different Frequencies of Oral Parafunction,” Journal of Prosthetic Dentistry 122, no. 2 (2019): 119–122. 30885582 10.1016/j.prosdent.2018.10.006

[14] R. Sava , N. Stanisic , L. Hindrot , et al., “Occlusal Acuity and Bite Force in Young Adults,” Neuroscience 568 (2025): 38–45. 39809359 10.1016/j.neuroscience.2025.01.024

[15] V. Donnarumma , R. Ohrbach , V. Simeon , F. Lobbezoo , N. Piscicelli , and A. Michelotti , “Association Between Waking‐State Oral Behaviours, According to the Oral Behaviors Checklist, and TMD Subgroups,” Journal of Oral Rehabilitation 48, no. 9 (2021): 996–1003. 34192368 10.1111/joor.13221 PMC8457156

[16] E. von Elm , D. G. Altman , M. Egger , et al., “The Strengthening the Reporting of Observational Studies in Epidemiology (STROBE) Statement: Guidelines for Reporting Observational Studies,” Journal of Clinical Epidemiology 61, no. 4 (2008): 344–349. 18313558 10.1016/j.jclinepi.2007.11.008

[17] E. Schiffman , R. Ohrbach , E. Truelove , et al., “International RDC/TMD Consortium Network International Association for Dental Research Orofacial Pain Special Interest Group, International Association for the Study of Pain Diagnostic Criteria for Temporomandibular Disorders (DC/TMD) for Clinical and Research Applications: Recommendations of the International RDC/TMD Consortium Network* and Orofacial Pain Special Interest Group†,” Journal of Oral & Facial Pain and Headache 28, no. 1 (2014): 6–27. 24482784 10.11607/jop.1151 PMC4478082

[18] I. Z. Alajbeg , E. Vrbanovic , I. Alajbeg , et al., “Time‐Course of Pain and Salivary Opiorphin Release in Response to Oral Capsaicin Differ in Burning Mouth Syndrome Patients, Temporomandibular Disorders Patients and Control Subjects,” Clinical Oral Investigations 28, no. 5 (2024): 246. 38589630 10.1007/s00784-024-05653-y

[19] M. Gikić , E. Vrbanović , M. Zlendić , and I. Z. Alajbeg , “Treatment Responses in Chronic Temporomandibular Patients Depending on the Treatment Modalities and Frequency of Parafunctional Behaviour,” Journal of Oral Rehabilitation 48, no. 7 (2021): 785–797. 33797785 10.1111/joor.13173

[20] M. Zlendić , E. Vrbanović , M. Tomljanović , K. Gall Trošelj , K. V. Đerfi , and I. Z. Alajbeg , “Association of Oral Behaviours and Psychological Factors With Selected Genotypes in Pain‐Related TMD,” Oral Diseases 30, no. 3 (2024): 1702–1715. 37036392 10.1111/odi.14583

[21] R. Ohrbach , Y. Gonzalez , T. List , A. Michelotti , and E. Schiffman , Diagnostic Criteria for Temporomandibular Disorders (DC/TMD) Clinical Examination Protocol: Version 02June2013 Dijagnostički kriteriji za temporomandibularne poremećaje (DK/TMP) Instrumenti procjene: Croatian Version 23March2021 Spalj S, Katic V, Alajbeg I, Celebic A. Trans. www.rdc-tmdinternational.org accessed on 18.09.2025.

[22] J. D. Greenspan , G. D. Slade , E. Bair , et al., “Pain Sensitivity Risk Factors for Chronic TMD: Descriptive Data and Empirically Identified Domains From the OPPERA Case Control Study,” Journal of Pain 12, no. 11 (2011): 61–74. 22074753 10.1016/j.jpain.2011.08.006 PMC3249228

[23] P. Svensson , T. List , and G. Hector , “Analysis of Stimulus‐Evoked Pain in Patients With Myofascial Temporomandibular Pain Disorders,” Pain 92, no. 3 (2001): 399–409. 11376913 10.1016/S0304-3959(01)00284-6

[24] M. D. Fox , A. Z. Snyder , J. L. Vincent , M. Corbetta , D. C. Van Essen , and M. E. Raichle , “The Human Brain Is Intrinsically Organized Into Dynamic, Anticorrelated Functional Networks,” Proceedings of the National Academy of Sciences of the United States of America 102, no. 27 (2005): 9673–9678. 15976020 10.1073/pnas.0504136102 PMC1157105

[25] M. Zlendić , E. Vrbanović , K. G. Trošelj , M. Tomljanović , K. V. Đerfi , and I. Z. Alajbeg , “Genetic Influence on Treatment Outcomes in Patients With Pain‐Related Temporomandibular Disorders,” Journal of Oral Rehabilitation 51, no. 8 (2024): 1542–1554. 38725226 10.1111/joor.13730

[26] C. Batailler , S. M. Brannon , P. E. Teas , and B. Gawronski , “A Signal Detection Approach to Understanding the Identification of Fake News,” Perspectives on Psychological Science 17, no. 1 (2022): 78–98. 34264150 10.1177/1745691620986135

[27] T. Nielson Hoberg , S. Frahm , K. Hennings , L. Arendt‐Nielsen , and C. Dahl Mørch , “Assessing the Modulation of Cutaneous Sensory Fiber Excitability Using a Fast Perception Threshold Tracking Technique,” Muscle & Nerve 60, no. 4 (2019): 367–375. 31107560 10.1002/mus.26520

[28] G. D. Slade , E. Bair , K. By , et al., “Study Methods, Recruitment, Sociodemographic Findings, and Demographic Representativeness in the OPPERA Study,” Journal of Pain 12, no. 11 (2011): 12–26. 10.1016/j.jpain.2011.08.001 PMC3666856 22074749

